# Combined Application of Negative Pressure Wound Therapy and Dermal Substitute for Coverage of a Complex Cutaneous Defect: A Case Report

**DOI:** 10.7759/cureus.88256

**Published:** 2025-07-18

**Authors:** Gladys M. Ballesteros Solís, Arym P Preza Estrada, Harvey Zamora-Veliz, José Luis Villarreal Salgado, Gerardo S Rea-Martínez

**Affiliations:** 1 Plastic and Reconstructive Surgery, Dr. Valentín Gómez Farías Regional Hospital, Instituto de Seguridad y Servicios Sociales de los Trabajadores del Estado (ISSSTE), Zapopan, MEX; 2 Plastic and Reconstructive Surgery, Dr. Valentín Gómez Farías Regional Hospital, Instituto de Seguridad y Servicios Sociales de los Trabajadores del Estado (ISSSTE), Guadalajara, MEX; 3 General Surgery, Hospital General, Instituto de Seguridad y Servicios Sociales de los Trabajadores del Estado (ISSSTE), La Paz, MEX; 4 Surgery, Instituto de Seguridad y Servicios Sociales de los Trabajadores del Estado (ISSSTE), Mazatlán, MEX; 5 Plastic and Reconstructive Surgery, Instituto de Seguridad y Servicios Sociales de los Trabajadores del Estado (ISSSTE), Guadalajara, MEX

**Keywords:** negative pressure system, split-thickness skin graft, upper extremity trauma, wound care management, wound reconstruction

## Abstract

Abrasion burns are a type of skin injury commonly associated with friction trauma, such as those caused by automobile accidents. These wounds, although superficial in appearance, can involve large areas of the integument and require surgical treatment to achieve adequate functional and aesthetic healing. We present the case of a female patient who suffered an extensive abrasion burn on her upper limb following a high-energy vehicle accident. The initial approach included surgical cleansing and placement of negative pressure therapy (vacuum-assisted closure system (VAC®)) to prepare the wound bed. This method allowed for exudate control, reduced bacterial load, and stimulated granulation tissue formation, all of which are necessary for subsequent reconstruction.

Once the recipient site was optimized, a full-thickness autologous skin graft was placed, which showed adequate integration, without infectious complications or signs of rejection. The postoperative course was favorable, with progressive functional recovery and satisfactory aesthetic results. The patient presented no complications during follow-up.

This case highlights the importance of using negative pressure therapy as an effective adjuvant in the management of complex acute wounds, allowing for better preparation of the recipient site and facilitating graft integration. It also emphasizes the need for a comprehensive and timely approach to the treatment of abrasion burns, especially in traumatic settings, where prompt intervention can directly influence clinical outcomes and patient quality of life. Early surgical management, combined with advanced wound care techniques, remains a cornerstone for optimizing recovery, minimizing scarring, and restoring limb function in patients with severe abrasion burns.

## Introduction

The most commonly used technique for covering a skin defect is skin autografting, with partial skin being more common. However, in certain areas, it is not only necessary to achieve stable coverage of the defect but also good skin pliability that allows the patient to perform their full range of motion [1]. Successful integration of split-thickness or deep skin grafts does not depend on a single factor, one of the most important being the graft's healing method [2]. The graft integration process occurs in three phases: (A) plasma inbibition, a process that lasts 48 to 72 hours during which the skin graft's nutrition depends on the absorption of exudate from the recipient bed; (B) blood vessel inoculation, where fibrin bridges form between the graft and the recipient bed, which subsequently form the microanastomoses of the capillaries; and (C) revascularization, in which new blood vessels grow from the recipient bed toward the graft; these, together with the existing vascular microanastomoses between the graft and the recipient bed, form the graft's definitive circulation [2]. To avoid complications secondary to the use of partial and deep skin grafts, different matrices have been developed that allow for dermal regeneration. Their use has expanded widely, from the treatment of burn patients to the coverage of lower limb defects [1]. Their fundamental indication has been and continues to be the improvement of the functional and cosmetic appearance of autograft coverage. However, for the incorporation of the regeneration matrix into the patient's bed, continuous and firm adherence is required. For this reason, to improve contact with the wound bed, various authors have used vacuum therapy. This involves applying a vacuum to the wound, homogenizing the pressure on the bed through the use of a polyurethane sponge. This allows for the evacuation of exudate and bacterial contamination, improves blood flow in the wound, and increases granulation tissue. Complex traumatic wounds sometimes require treatment with large flaps and prolonged hospital stays. Currently, the use of dermal substitutes combined with vacuum therapy allows for improved quality of the results obtained in a shorter time [2].

## Case presentation

**Figure 1:**
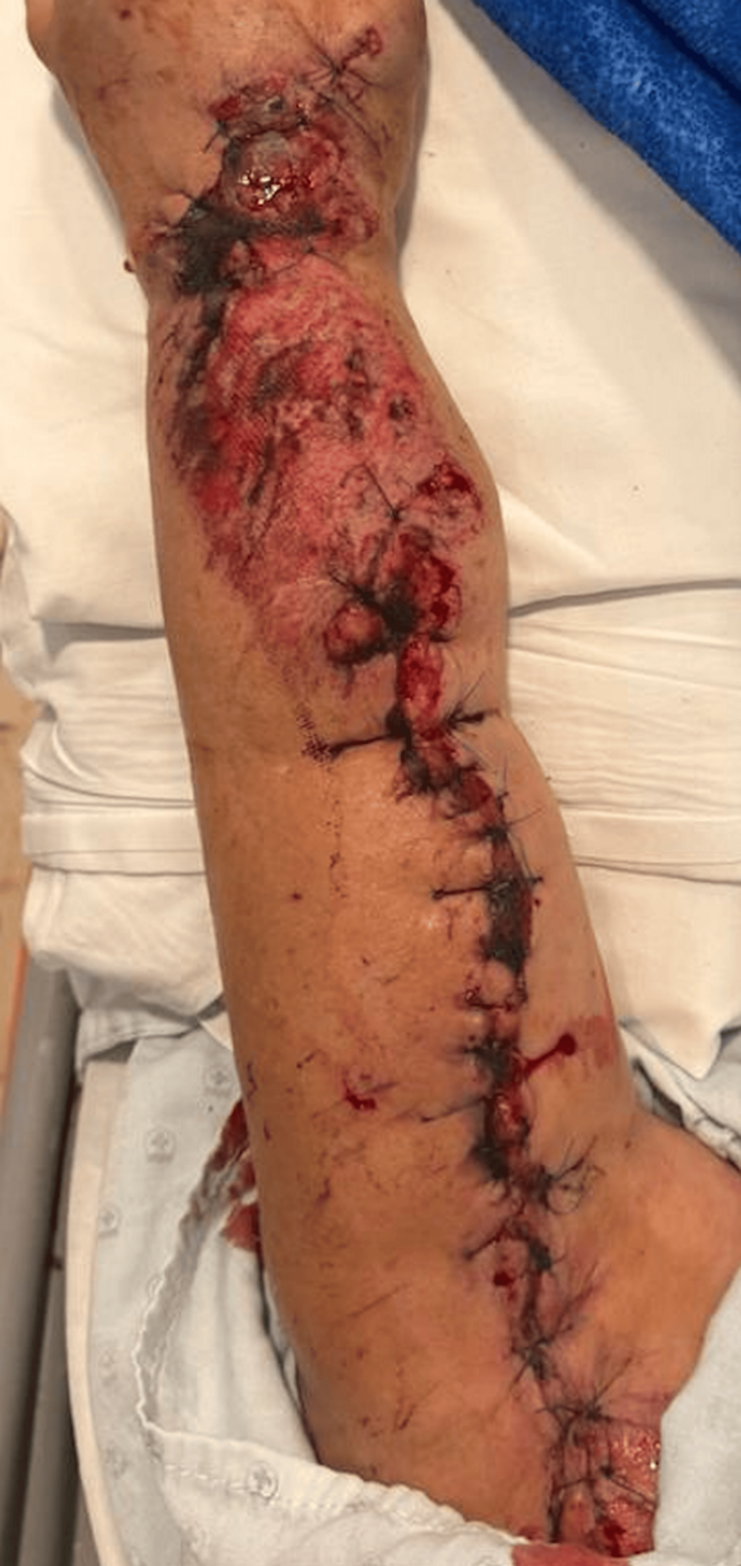
Forearm closure with continuous stitches

This is a 66-year-old female patient who suffered multiple injuries secondary to a bus accident in which a moving train collided, causing multiple wounds throughout her body. Physical examination revealed a wound in the area of ​​her left forearm measuring approximately 30 cm with exposed bone. She was urgently transferred to a medical unit for treatment, where she underwent surgical lavage and wound closure with continuous 4-0 nylon sutures (Figure 1). 

**Figure 2:**
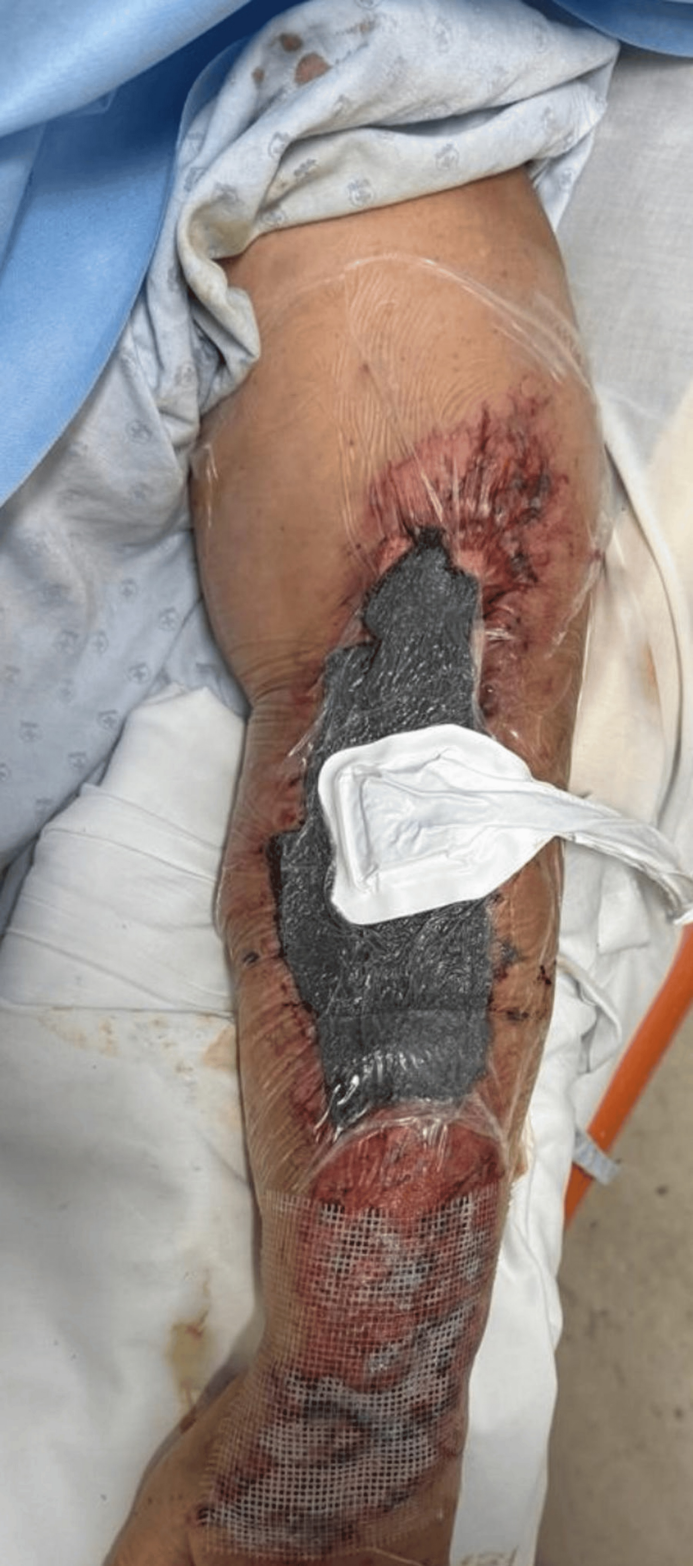
Vacuum-assisted closure system (VAC therapy)

The patient was transferred to our hospital unit for evaluation, where the decision was made to place a vacuum-assisted closure system (VAC therapy) as part of the initial wound management, with no complications occurring during its application (Figure 2).

**Figure 3:**
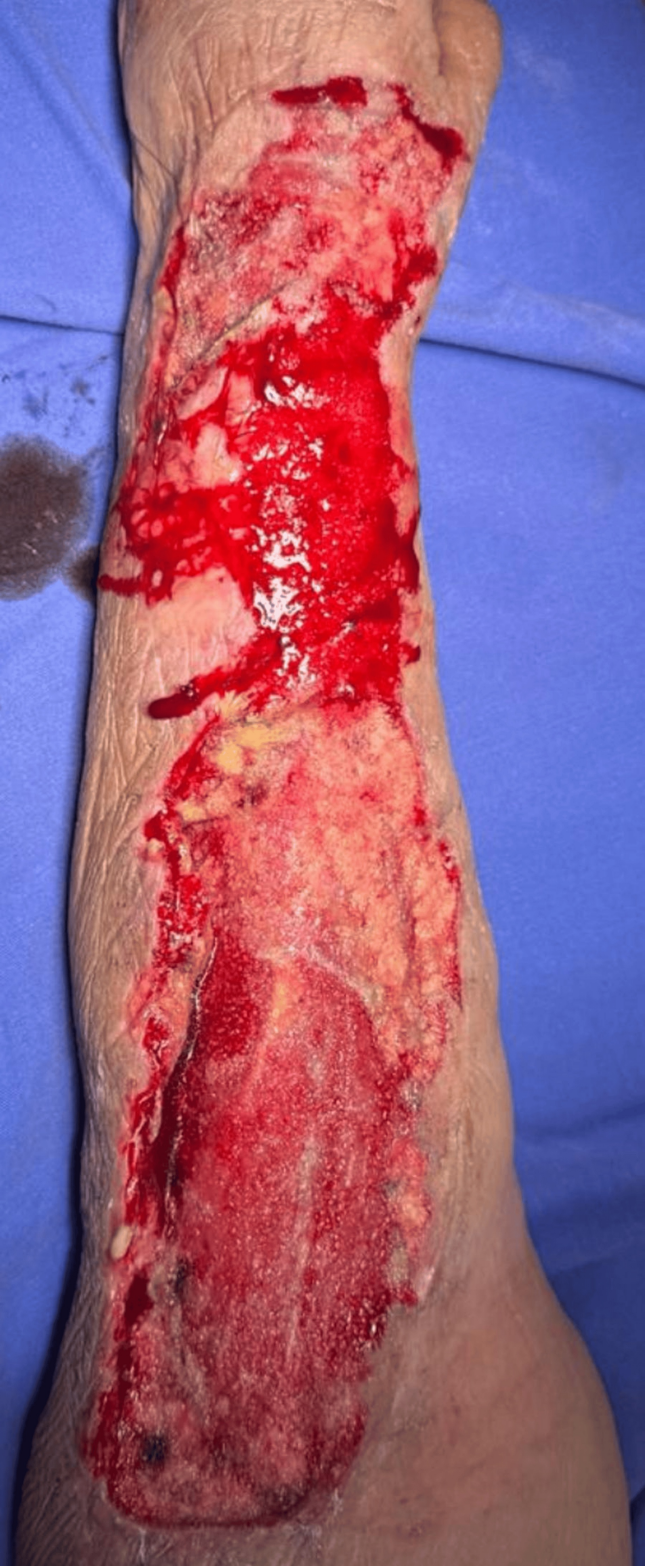
Removal of the VAC system for skin graft placement VAC: vacuum-assisted closure system

**Figure 4:**
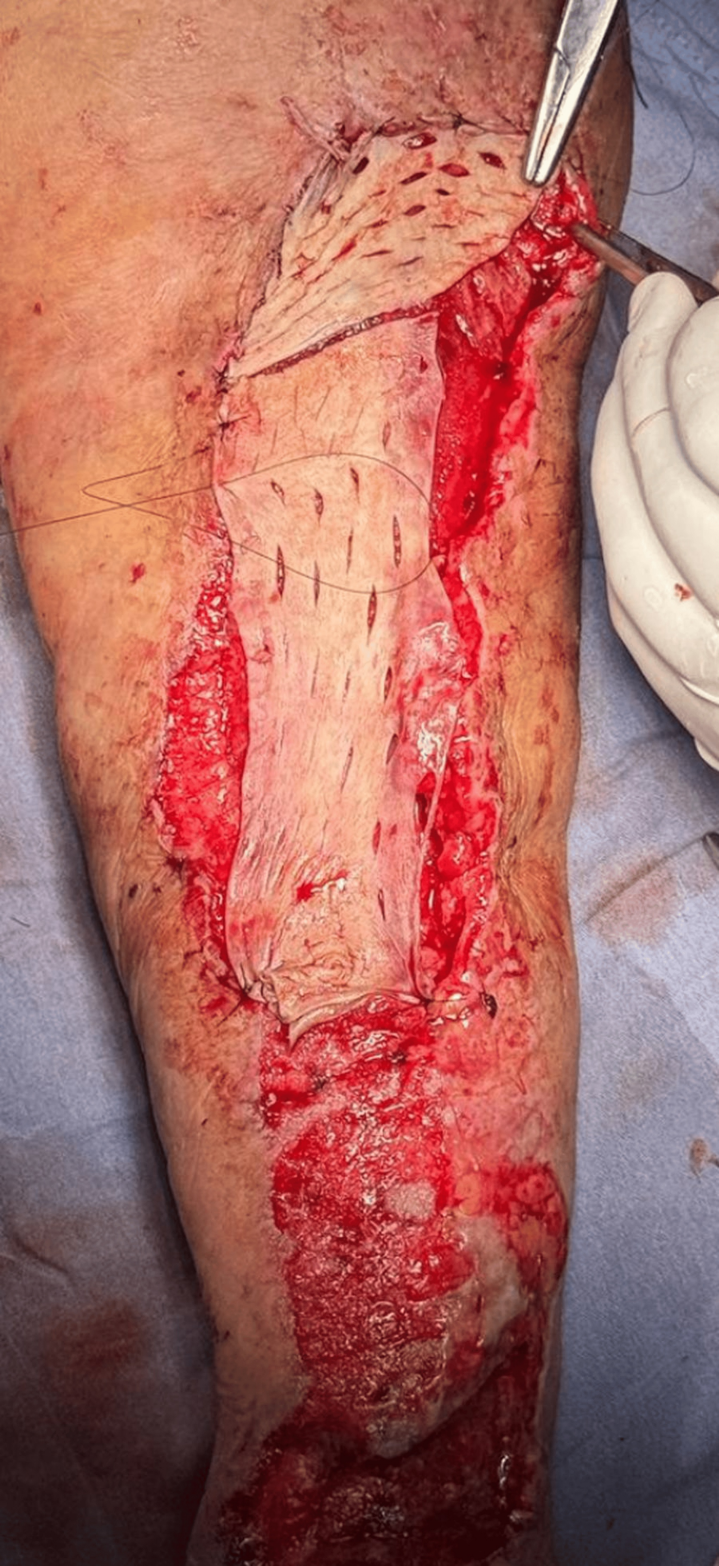
Skin graft placement

After six days, the device was replaced as scheduled, maintaining adequate clinical progress. Skin graft placement was scheduled for six days later. During surgery, surgical cleansing of the left upper extremity was performed, removing biofilm and necrotic tissue (Figure 3). Subsequently, full-thickness skin graft placement was indicated. A Zimmer dermatome was used to harvest the graft from the anterior portion of the left thigh, followed by the placement of a petroleum jelly dressing over the donor area (Figure 4). 

The graft was meshed in a 3:1 ratio and secured to the recipient site with surgical staples and 3-0 nylon sutures. A Jelonet dressing was applied, and the limb was immobilized with a splint and posterior bandage to protect and secure the graft. During immediate postoperative follow-up, no complications of the surgical wound were observed.

Postoperatively, the patient progressed favorably with no acute complications resulting from the procedure. Therefore, she was discharged 17 days after surgery, with a prescription for analgesics and antibiotics. She was discharged with a follow-up appointment in five days.

**Figure 5:**
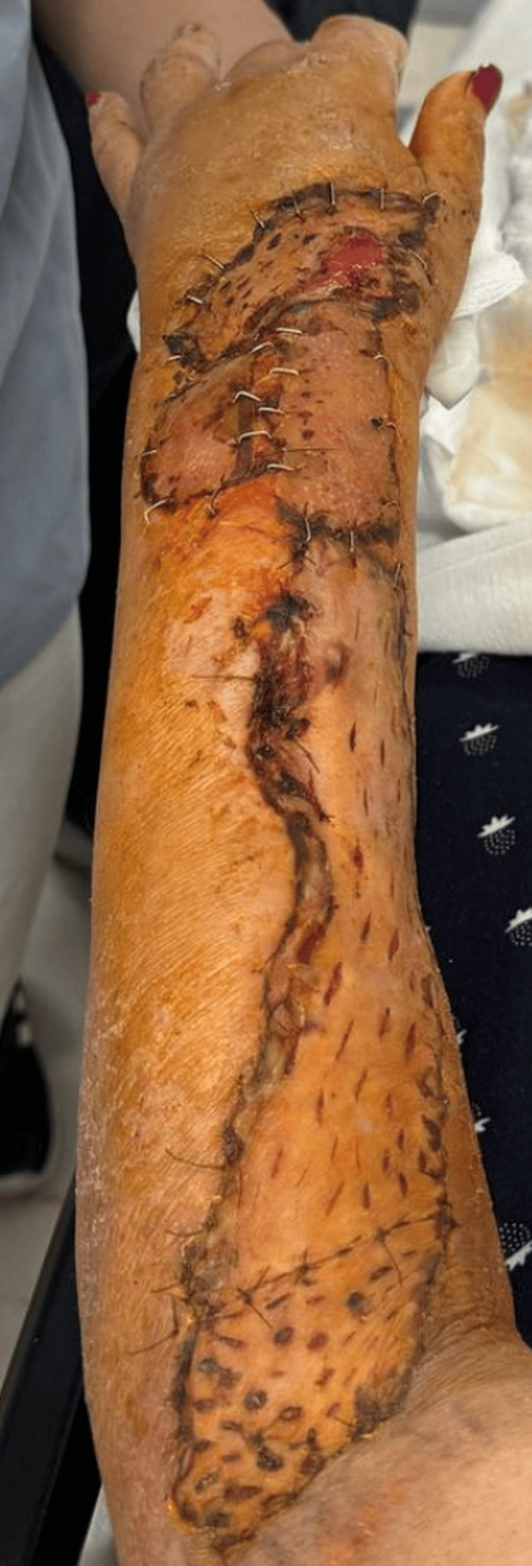
Subsequent postoperative evaluation (five days later)

**Figure 6:**
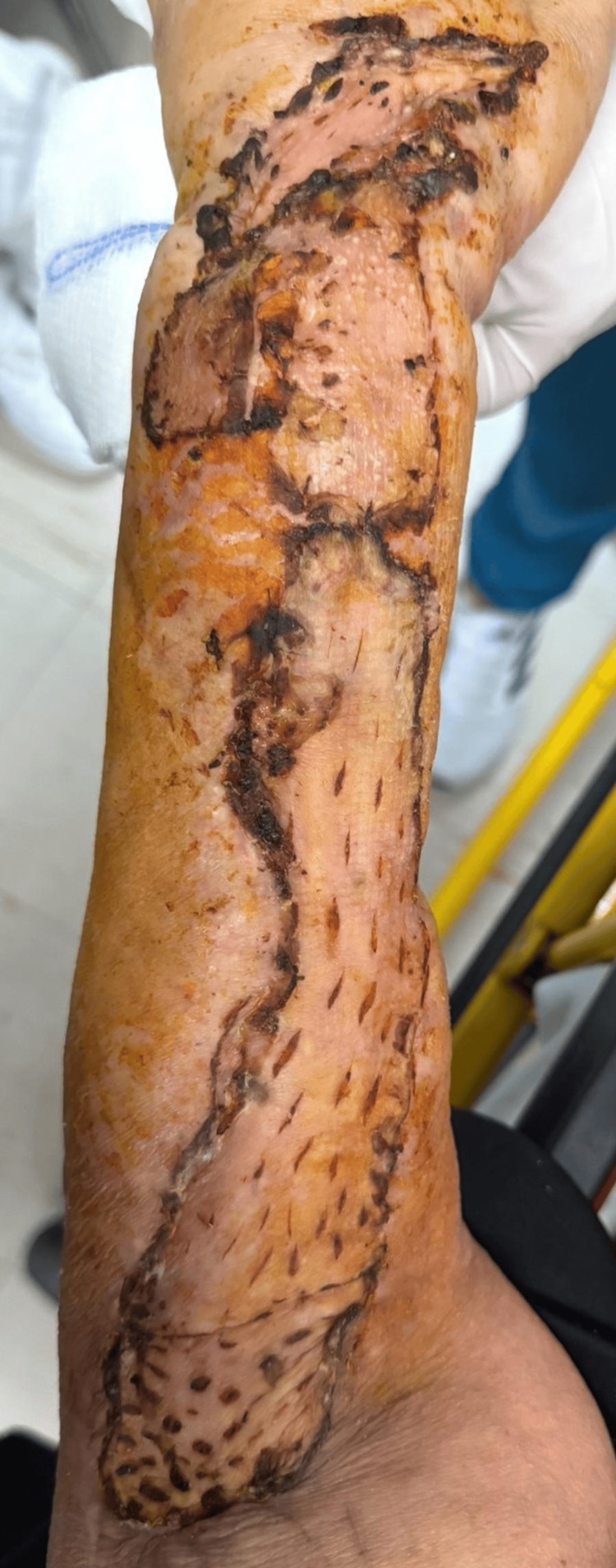
Postoperative evaluation (12 days later)

At the subsequent postoperative evaluation (five days later), the graft was found to be well-vascularized, with well-adhered edges and no signs of local complications (Figure 5). The wound was treated, and a follow-up appointment was scheduled in seven days, during which adequate healing was observed without signs of infection or complications (Figure 6).

## Discussion

Negative pressure wound therapy (NPWT) has been used since 1989. It is currently presented as an alternative and accessible method in any hospital setting, effective in preparing acute and chronic wounds for primary, delayed primary, or secondary closure. It is gradually gaining acceptance among physicians in our country. NPWT is a mechanical treatment used as an adjuvant in wound healing; it can be applied continuously or intermittently, for both acute and chronic wounds [3]. The vacuum-assisted wound closure system is based on the use of negative pressure. Subatmospheric pressure is created at the wound site by previously placing a polyurethane sponge inside the wound edges and covering it tightly with self-adhesive plastic. A small incision is made in the plastic over the sponge and a suction tube is placed, which is in turn connected to an automatically controlled mechanical pump, which by applying suction creates an airtight seal that protects the wound, drains fluids through the pores of the sponge and brings the edges of the wound closer together, accelerating the healing process [4].

Currently, accidents cause various injuries, and in many cases, the use of partial skin grafts for coverage is necessary as part of a reconstructive technique [5]. Grafts represent a major challenge for plastic surgeons due to the scarring that results in the donor areas [5]. The normal healing process lasts approximately six months. In the initial phases, it is important to use different types of dressings, such as xenografts, autografts, synthetic dressings, etc., in the partial skin donor area. The objective is to find the one that provides the greatest benefit in the quality of healing and facilitates adequate recovery of the donor area in affected patients [5].

Grafts can be categorized in four main ways. One method classifies them by histological composition: simple or free grafts consist of a single tissue type, while composite grafts are made up of two or more types of tissue. Another classification depends on the donor source. Autografts originate from the same individual who will receive the graft; allografts (or homologous grafts) come from a different individual of the same species; and xenografts (or heterologous grafts) involve a donor and recipient from different species. A third approach focuses on thickness. Full-thickness grafts include both the epidermis and the entire dermis, along with skin appendages. Split-thickness grafts contain the epidermis and a portion of the dermis and are further divided into thin, intermediate, and thick, depending on how much dermis is included. Lastly, grafts may also be classified by processing technique. After being harvested, they can be modified to cover a larger area. This is done either in the operating room using a mesher to create a meshlike pattern (mesh grafts) or in the lab through cell culture expansion. The specific clinical context will guide the selection of the most appropriate graft type [6]. The clinical situation determines the type of graft used.

The successful integration of autologous skin grafts depends primarily on three clinical factors: adhesion, perfusion, and viability, all of which are directly linked to the graft’s vascularization. In the first 24 hours following transplantation, the graft absorbs plasma from the recipient site, which forms a fibrin matrix that anchors and nourishes the graft, a process known as the plasma inosculation phase. Subsequently, small capillaries connect the graft with the wound bed through microvascular anastomosis (the inosculation phase), although at this stage, adhesion remains fragile and may show signs of cyanosis. The formation and expansion of new blood vessels are essential for graft survival, with true blood perfusion typically established between postoperative days five and seven, marking the revascularization phase. Once the graft becomes stable in the recipient site, usually after day 10, contractile myofibroblasts and structural proteins contribute to graft contraction, a process that can extend over several months and potentially impact cosmetic outcomes. Myofibroblasts, specialized fibroblasts from granulation tissue, develop features resembling smooth muscle cells, such as microfilaments and alpha-smooth muscle actin expression. Beyond contraction, they also play a role in synthesizing components of the extracellular matrix. The extent to which grafts influence the wound's myofibroblast population depends on dermal thickness: Thicker grafts tend to contain fewer myofibroblasts, resulting in reduced tissue contraction [6].

Fibronectin, which serves as a key anchoring component in the provisional extracellular matrix, is gradually lost as collagen fibers are laid down. In full-thickness grafts, fibronectin tends to be less prominent and disappears earlier compared to its presence in split-thickness grafts. Interestingly, superficial wounds treated with full-thickness skin showed more contraction than those covered with split-thickness grafts. Conversely, in deeper wounds extending to the muscular fascia, grafts composed solely of epidermis or split-thickness skin resulted in greater contraction than those covered with full-thickness skin. These findings highlight the importance of matching the graft's thickness to the depth of the wound to optimize healing and minimize undesirable contraction [6].

The use of full-thickness skin grafts remains a fundamental tool in skin reconstruction, especially for traumatic injuries such as abrasion burns. The combination with NPWT has proven to be an effective strategy for optimizing the recipient site, promoting graft adherence, vascularization, and viability. This approach not only accelerates healing and reduces complications but also improves functional and aesthetic outcomes for the patient. Appropriate selection of the graft type based on the depth and characteristics of the wound, as well as meticulous preparation of the recipient site, are key elements for therapeutic success. As these technologies become increasingly integrated into clinical practice, their understanding and proper application will contribute to improving the quality of surgical management of complex wounds in diverse hospital settings.

## Conclusions

The comprehensive management of abrasion burns secondary to motor vehicle accidents requires a timely, multidisciplinary approach. In this case, the early application of a negative pressure-assisted closure (VAC) system optimized the wound bed, controlling exudation, reducing bacterial load, and promoting granulation tissue formation. Subsequent full-thickness autologous skin grafting resulted in adequate graft integration and a favorable outcome without complications. This case highlights the value of negative pressure therapy as an effective therapeutic bridge to definitive reconstruction in patients with extensive abrasion injuries.
